# Power- and Low-Resistance-State-Dependent, Bipolar Reset-Switching Transitions in SiN-Based Resistive Random-Access Memory

**DOI:** 10.1186/s11671-016-1572-9

**Published:** 2016-08-12

**Authors:** Sungjun Kim, Byung-Gook Park

**Affiliations:** Department of Electrical and Computer Engineering, Inter-University Semiconductor Research Center (ISRC), Seoul National University, Seoul, 08826 South Korea

## Abstract

A study on the bipolar-resistive switching of an Ni/SiN/Si-based resistive random-access memory (RRAM) device shows that the influences of the reset power and the resistance value of the low-resistance state (LRS) on the reset-switching transitions are strong. For a low LRS with a large conducting path, the sharp reset switching, which requires a high reset power (>7 mW), was observed, whereas for a high LRS with small multiple-conducting paths, the step-by-step reset switching with a low reset power (<7 mW) was observed. The attainment of higher nonlinear current-voltage (*I-V*) characteristics in terms of the step-by-step reset switching is due to the steep current-increased region of the trap-controlled space charge-limited current (SCLC) model. A multilevel cell (MLC) operation, for which the reset stop voltage (*V*_STOP_) is used in the DC sweep mode and an incremental amplitude is used in the pulse mode for the step-by-step reset switching, is demonstrated here. The results of the present study suggest that well-controlled conducting paths in a SiN-based RRAM device, which are not too strong and not too weak, offer considerable potential for the realization of low-power and high-density crossbar-array applications.

## Background

Conventional memories such as dynamic random-access memory (DRAM) and NAND flash memory that are based on charge storage have been utilized in many applications until now, and the continually increasing demand for these memories is a response to the era of big data and the Internet of Things (IoT) [1, 2]. In recent years, however, and as expected, a near-future occurrence of the physical limits of these commercial memories has been identified. As an alternative, new approaches such as three-dimensional (3D) structure technology, multilevel cell (MLC) technology, and emerging memory technology have been widely researched [3–5]. Among the emerging memories, the resistive random-access memory (RRAM) has attracted considerable interest due to numerous advantages that include a simple structure, a fast switching speed, a low-voltage operation, an extendibility to the 3D structure, and an MLC operation [6–31]. Although extensive research has been carried out on the oxide-based RRAM that is a strong candidate for nonvolatile memory applications, it is also worth investigating a nitride-based RRAM with a silicon bottom electrode (BE), as it has a complete compatibility with the standard complementary metal-oxide-semiconductor (CMOS) processing [26–31]. So far, however, only a small amount of discussion regarding the resistive-switching characteristics of the nitride-based RRAM has occurred.

In particular, MLC is an important key technology for high-density nonvolatile memories and synaptic devices, as it overcomes the limits of conventional lithography [32]. In the RRAM, the MLC can be easily achieved through a controlling of the stop voltage (*V*_STOP_) for reset switching. The main aim of this study is a clarification of the switching parameters to differentiate the three types of reset-switching transition in terms of a fabricated Ni/Si_3_N_4_/*p*^*+*^-Si-based RRAM device.

## Methods

The sample was prepared as follows: After the standard cleaning processes, a *p*^+^ Si BE was highly doped using a BF_2_^+^ ion implanting with a dose of 5 × 10^15^ cm^−2^ (converted to a peak concentration of 10^20^ cm^−3^). A SiN of 4.1 nm thickness was then deposited on the implanted silicon wafer using low-pressure chemical vapor deposition (LPCVD) at 785 °C after an annealing process was performed at 1050 °C for 10 min; subsequently, the Ni top electrode (TE) was deposited and patterned through a shadow mask that contains circular patterns with a 100-μm radii. All of the electrical properties were characterized at room temperature according to the DC voltage sweep mode and the pulse mode using the Keithley 4200-SCS semiconductor parameter analyzer (SPA) and the 4225-PMU ultra-fast *I-V* module, respectively. Also, the *p*^*+*^-Si BE was grounded and control biases were applied to the Ni TE over the measurements.

## Results and Discussion

**Fig. 1:**
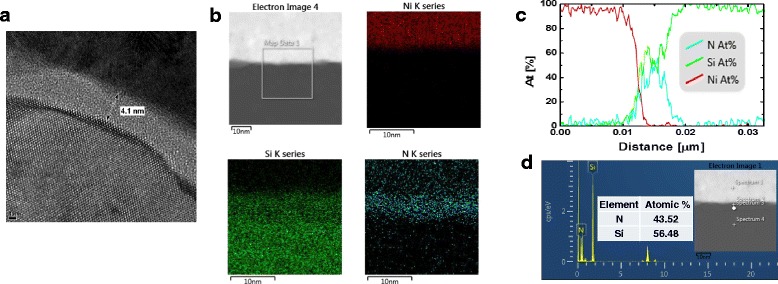
Transmission electron microscopy (TEM) and energy-dispersive X-ray spectroscopy (EDS) analysis. **a** Cross-sectional TEM image of Ni/SiN_*x*_/Si structure. **b** STEM image and EDS maps of distribution Ni, Si, and N. **c** EDS line profile of Ni/SiN_*x*_/Si structure. **d** EDS spectrum of SiN_*x*_ layer

The thickness of SiN_*x*_ layer was confirmed using cross-sectional transmission electron microscopy (TEM) image of Ni/SiN_*x*_/Si structure as shown in Fig. 1a. Scanning TEM (STEM) image and energy-dispersive X-ray spectroscopy (EDS) maps of distribution Ni, Si, and N is shown in Fig. 1b. Atomic percentage of each layer was confirmed by EDS line profile and spectrum of Ni/SiN_*x*_/Si structure as shown in Fig. 1c, d, respectively.

**Fig. 2:**
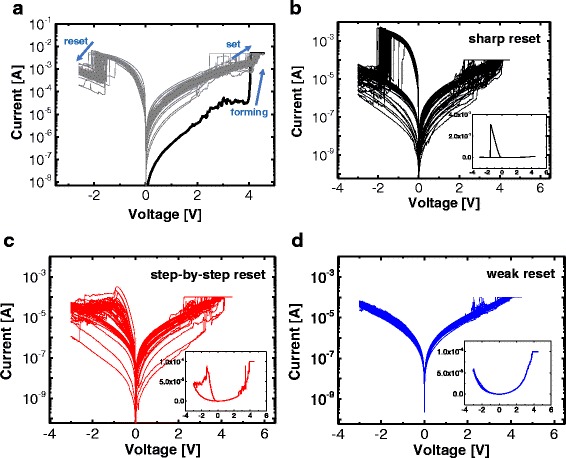
*I-V* characteristics of the Ni/Si_3_N_4_/*p* ^*+*^-Si RRAM device. **a** Sharp reset transition with CC of 5 mA. **b** Sharp reset transition with CC of 100 μA. **c** Step-by-step-reset transition with CC of 100 μA. **d** Weak reset transition with CC of 100 μA

Figure 2a shows the bipolar current-voltage (*I-V*) characteristics of the Ni/Si_3_N_4_/*p*^*+*^-Si RRAM device under the DC voltage sweep mode. After the positive electroforming process (a thick black line), a current compliance (CC) of 5 mA was used to activate a pristine cell with a high initial resistance; here, the CC is used to protect the device from a permanent dielectric breakdown. A negative voltage sweep was conducted without the CC for the reset process that performs a switch from the low-resistance state (LRS) to the high-resistance state (HRS). A positive sweep switches the device back to the LRS for the reset switching. The Ni/Si_3_N_4_/*p*^*+*^-Si RRAM device then showed a forming-less process, indicating that the forming voltage is within the distribution range of the set voltage; notably, with the 5-mA CC, sharp current drops were observed for all of the reset switching during 100 consecutive DC voltage sweeps. Alternatively, the set switching, whereby the current is increased sharply or gradually, is more complicated; also, for some of the switching, the set process was completed after a gradual current drop due to an incomplete reset switching, suggesting that the set and the reset switching are strongly affected by the previous switching events.

**Fig. 3:**
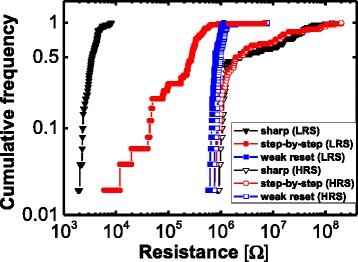
Cumulative frequency of the LRS and HRS resistances for the three types of reset switching

Next, the reset-switching behaviors under a 100-μA CC were investigated. According to the *I-V* characteristics that are shown in Fig. 2b–d, reset switching can be classified into three types. Figure 2b shows a sharp reset switching that occurs even though a low CC of 100 μA was set for the device. The low LRS resistance and high reset current (*I*_RESET_) are caused by a current overshoot that is for the set process. The main reason for the occurrence of the current overshoot is an instrument-response time that is slower than the switching time of the device. Figure 2c exhibits a step-by-step reset transition that starts with a relatively high LRS resistance; here, the comparability of the *I*_RESET_ to the CC of 100 μA is probably due to a reduced overshoot effect that occurs during the set process. Figure 2d shows a weak reset switching, where the current drop is within only a few microamperes and the LRS resistance is very slightly changed; here, it is likely that the weakness is due to an incomplete formation of the conducting path in terms of the set process. The current overshoot effect in the proposed fabricated device occurs randomly, and this causes a large variation of the LRS [33, 34]. If appropriate actions such as a series resistance and a transistor were prepared for the device to reduce the current overshoot, the CC could control the LRS resistance and the reset current more efficiently [35, 36]; however, the provision of a clue to gain an understanding of the reset-transition behaviors in the LRS itself is worthwhile. Figure 3 shows the cumulative frequency of the LRS and HRS resistances for the three types of reset switching. The distribution of the sharp reset switching is less dispersive compared with the distribution of the step-by-step reset switching. For the weak reset switching, only a slight difference is evident between the LRS and the HRS. According to the findings of this paper, a proper set overshoot is needed to obtain an effective resistive-switching behavior.

**Fig. 4:**
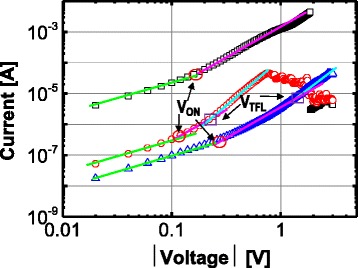
Double logarithmic plots of *I-V* curve in the LRS for the three types of reset switching

Through the double logarithmic plots of the *I-V* curve in the LRS, a preliminary estimation of the conducting mechanism of the fabricated device was made. The current transport is governed by a power law (i.e., *I~V*^*m*^), but with different slopes, as shown in Fig. 4. The ohmic behavior (*I~V*) was changed to Child’s square law (*I~V*^2^) for the three types of switching, and a higher slope region (*I~V*^*m*^, *m* > 2) was observed for the switchings with the step-by-step and the weak reset transitions. This conduction can be explained by the trap-controlled space charge limited current (SCLC) theory [37–41]. The trap sites that are composed of the dangling bonds in the trivalent Si atoms can be used as a medium through the trapping and de-trapping of the carriers. The ohmic conduction in a low-voltage region is dominated by free electrons that are thermally dominated when they are compared to the injected electrons. The injected electrons from the electrode become especially predominant with the increasing of the voltage at the *V*_ON_ that can be expressed as follows:

$$ {V}_{\mathrm{ON}}=\left[8/9\left(\theta +{\theta}_t\right)/\theta \right]\cdot \left[q{n}_0{L}^2/{\varepsilon}_r{\varepsilon}_0\right], $$

where *θ* is the free carrier density, *θ*_*t*_ is the concentration of the traps, *q* is the electron charge, *n*_*0*_ is the thermally produced free-electron density, *L* is the nitride thickness, *ε*_*r*_ is the static-dielectric constant, and *ε*_*0*_ is the permittivity of the free space. The trap-unfilled SCLC is changed to a trap-filled SCLC at the trap-filled limited voltage (*V*_TFL_), thereby resulting in a further slope increase. The sharp reset switching was finished before the *V*_TFL_ was reached, while the switchings with the step-by-step reset and the weak reset were completed after the *V*_TFL_ was reached.

**Fig. 5:**
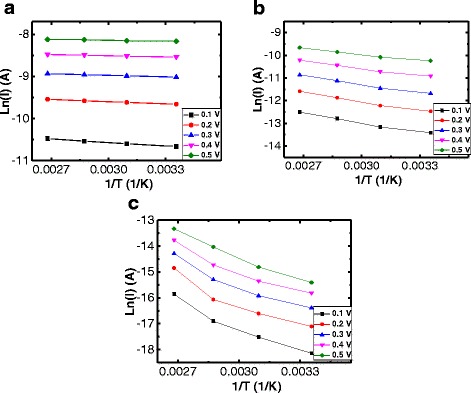
Plot of ln(*I*) vs. 1/*T* at low-voltage region (−0.1 to −0.5 V) for the LRS of the three types of reset switching (**a**–**c**)

**Fig. 6:**
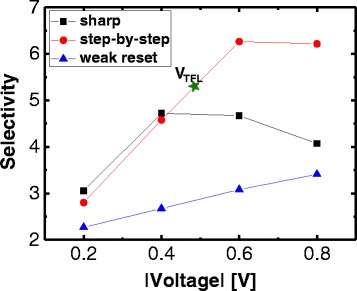
Nonlinearity as a function of the read voltage of the three types of reset switching in the LRS

Unlike the conventional metal-oxide-based RRAM devices such as NiO, TiO_2_, and CuO_2_, for which a metallic ohmic conduction with a slope of 1 in the log-log scale is shown, SiN_*x*_-based RRAM devices exhibited nonlinear *I-V* curves in the LRS. The nonlinear resistive switching in the LRS is one of the major strong points in the two-terminal-based crosspoint array application. The earlier report for which the Ni/SiN/TiN RRAM stack is used confirmed this intrinsic nonlinear characteristic, which is attributed to the different trap-assisted tunneling mechanism that applies in this case; here, the nonohmic *I-V* curves of the Ni/SiN/*p*^*+*^-Si RRAM device can also be explained by the different properties of the conducting path in the LRS. The metallic conducting paths of the metal-oxide-based RRAM that are in the LRS are formed by oxygen vacancies. In this case, the resistance is increased when the temperature is elevated; contrarily, the SiN-based RRAM employs the previously mentioned trap-related conduction mechanism that ensures the production of a nonlinear *I-V* curve without the use of an extra selection device. Figure 5a–c shows the plot of the ln(*I*) vs. the 1/*T* in the low-voltage region (−0.1 to −0.5 V) for the LRS of the three types of reset switching. A negative slope indicates that the underlying conduction mechanism is based on a trap-to-trap hopping that occurs through nitride vacancies, rather than the intervention of the metallic conducting filaments. Figure 6 shows the nonlinearity of the three types of reset switching in the LRS. The selectivity can be defined as the ratio of the current at the read voltage (*V*_READ_) to that at half of the *V*_READ_ (1/2 *V*_READ_). The selectivity of the step-by-step reset switching is higher than that of the sharp reset switching by more than the absolute value of 0.4 V, and this is because the steep increased current in the high-voltage region after the *V*_TFL_ of 0.5 V is reached leads to a higher nonlinearity for the step-by-step reset switching.

**Fig. 7:**
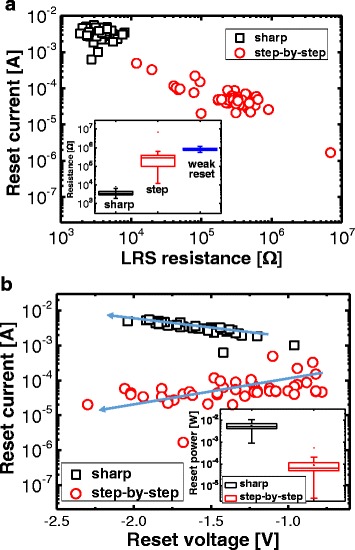
**a** *I*_RESET_ as a function of the LRS resistance and **b** *I*_RESET_ as a function of the *V*_RESET_

As part of the authors’ determination of the reset-transition behaviors, a further investigation of the switching parameters was conducted, whereby the relationship between the resistive-switching parameters was closely examined. Figure 7a shows the *I*_RESET_ as a function of the LRS resistance in terms of the switchings with the sharp and the step-by-step resets. The sharp-reset-switching relationship between the two parameters is not clear; alternatively, for the switching with the step-by-step reset, the *I*_RESET_ is decreased with an increasing of the LRS resistance. Notably, the three reset types are determined by the LRS resistance in the inset of Fig. 7a, indicating that, for the step-by-step reset switching, the conducting paths are carefully controlled for the set switching. Figure 7b shows the scatter plot of the *I*_RESET_ vs. *V*_RESET_ in terms of the switchings with the sharp and the step-by-step resets. The *I*_RESET_ is directly proportional to the absolute *V*_RESET_ value for the sharp and the step-by-step resets; meanwhile, the step-by-step reset shows the opposite trend. The inset of Fig. 7b shows the distribution of the reset power (*P*_RESET_) of the switchings with respect to both the sharp and the step-by-step resets. The two reset transitions are clearly distinguished by an *I*_RESET_ of 7 mA and a *P*_RESET_ of 1 mW.

**Fig. 8:**
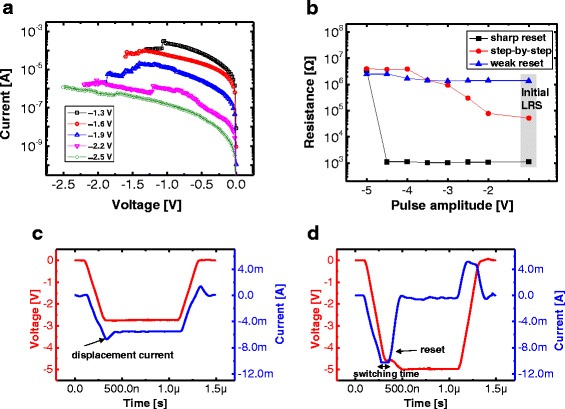
**a** Multilevel *I-V* characteristics of the reset process for step-by-step reset switching. **b** Resistance value after different pulse amplitudes from −2 to −5 V are applied to the devices. Applied voltage pulse (−3 **c** and −5 V **d**) and transient current response for sharp reset switching

To evaluate the MLC capability, the DC characteristics of the Ni/SiN/*p*^*+*^-Si device were investigated through a controlling of the *V*_STOP_. Figure 8a shows the multilevel *I-V* characteristics of the reset process for the step-by-step reset switching. The current levels of the multiple states were gradually decreased by more than three orders of magnitude, while the *V*_STOP_ was increased from −1.3 to −2.5 V; moreover, the pulse characteristics were checked for the three switching types. Figure 8b shows the resistance value after the different pulse amplitudes from −2 to −5 V were applied to the devices; for the abrupt reset switching, a switching response was not registered until −4.5 V and a sharp current drop was observed at −5 V. For the step-by-step reset switching, an initially higher LRS resistance is required for the employment of the MLC operation; however, a weak resistance change was obtained for the initially very high LRS resistance that is caused by the set switching. It is therefore important to ensure a proper LRS resistance range after the set switching for the MLC operation. Figure 8c, d shows the applied voltage pulse (−3 and −5 V) and the transient current response for the sharp reset switching. A significant current change was not recorded during the peak voltage of −3 V after the occurrence of an external displacement current, which is caused by the capacitive charging effect in the test system that is shown in Fig. 8c; alternatively, a distinctive current drop was observed when the peak voltage of −5 V was applied to the device.

**Fig. 9:**
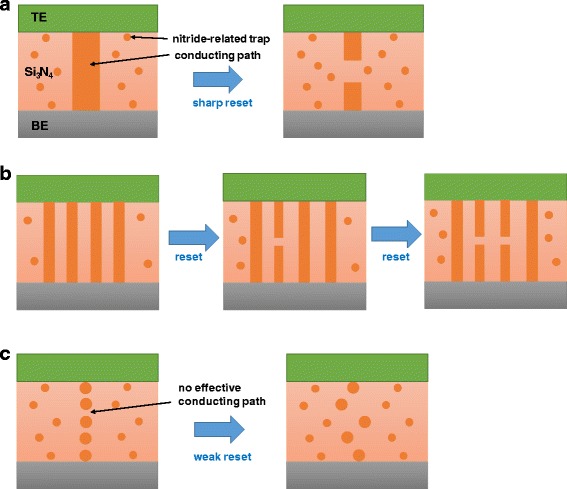
Schematics showing possible reset-switching models for the three types of reset-switching behavior: **a** sharp reset, **b** step-by-step reset, and **c** weak reset

Based on the electrical measurement results, a possible reset-switching model is proposed in this paper for the three types of reset-switching behavior, as shown in Fig. 9a–c. The conducting path may be made up of abundant traps which originate from dangling bonds in the trivalent Si atoms. Here, we explain the switching and conduction mechanism for three reset switching transitions by using conduction path model. Three reset-switching transitions can be explained by the size of conducting paths, which is related to the amount of traps [42]. For the sharp reset switching, however, a singly connected large conducting path could be ruptured; that is, the sharp reset process that is caused by the electrical field with thermal assistance turns to the HRS, as shown in Fig. 9a. For the step-by-step reset switching, the weakest part among the multiple conducting paths would be ruptured first, followed by the additional rupture processes, as shown in Fig. 9b. For a weak reset switching, initially weak conducting paths would be formed, followed by the absence of an effective reset process, as shown in Fig. 9c.

## Conclusions

The different bipolar reset transitions in terms of an Ni/SiN/Si RRAM device were investigated in this study depending on the reset power and the resistance value of the LRS, which is determined by the forming and set processes. The sharp reset switching was observed for the high reset power (>7 mW) and the low LRS, whereas the step-by-step reset switching was observed for the low reset power (<7 mW) and the high LRS. Higher nonlinear *I-V* characteristics and a gradual reset change regarding the step-by-step reset switching would be two of the most virtuous merits of the low-power and high-density crossbar array.
